# Potential Effects of Nutraceuticals in Retinopathy of Prematurity

**DOI:** 10.3390/life11020079

**Published:** 2021-01-22

**Authors:** Jessica K. W. Tsang, Susanne A. Wolf, Inga M. Pompoes, Antonia M. Joussen, Wai Ching Lam, Di Yang, Amy C. Y. Lo

**Affiliations:** 1 Department of Ophthalmology, Li Ka Shing Faculty of Medicine, The University of Hong Kong, Hong Kong, China; u3004548@connect.hku.hk (J.K.W.T.); waichlam@hku.hk (W.C.L.); 2 Department of Ophthalmology, Charité–University Medicine Berlin, 10117 Berlin, Germany; susanne.wolf@charite.de (S.A.W.); inga.pompoes@charite.de (I.M.P.); antonia.joussen@charite.de (A.M.J.); 3 Department of Ophthalmology, First Affiliated Hospital of Kunming Medical University, Kunming Medical University, Kunming 650500, China

**Keywords:** carotenoid, flavonoid, herbal extracts, inflammation, oil, preterm, retinal neovascularization, VEGF

## Abstract

Retinopathy of prematurity (ROP), the most common cause of childhood blindness, is a hypoxia-induced eye disease characterized by retinal neovascularization. In the normal retina, a well-organized vascular network provides oxygen and nutrients as energy sources to maintain a normal visual function; however, it is disrupted when pathological angiogenesis is induced in ROP patients. Under hypoxia, inadequate oxygen and energy supply lead to oxidative stress and stimulate neovasculature formation as well as affecting the function of photoreceptors. In order to meet the metabolic needs in the developing retina, protection against abnormal vascular formation is one way to manage ROP. Although current treatments provide beneficial effects in reducing the severity of ROP, these invasive therapies may also induce life-long consequences such as systemic structural and functional complications as well as neurodevelopment disruption in the developing infants. Nutritional supplements for the newborns are a novel concept for restoring energy supply by protecting the retinal vasculature and may lead to better ROP management. Nutraceuticals are provided in a non-invasive manner without the developmental side effects associated with current treatments. These nutraceuticals have been investigated through various in vitro and in vivo methods and are indicated to protect retinal vasculature. Here, we reviewed and discussed how the use of these nutraceuticals may be beneficial in ROP prevention and management.

## 1. Introduction

Retinopathy of prematurity (ROP) is a major cause of blindness in children worldwide. It is the most prevalent retinal disease in the developing countries [1]. Although childhood blindness is relatively uncommon and contributes to around 4% of total cases of blindness, vision loss or impairment imposes a dramatic impact on the development of children, especially neurodevelopment outcomes, who have to live without vision or endure severe threat to vision during their growth and the rest of their life [1,2].

In the 19th century, premature infants had a high mortality rate, which is mainly caused by hypothermia, infection, and respiratory distress [3,4]. An oxygen chamber was invented to provide supplemental oxygen for the postnatal development of preterm babies and thus increased their survival. However, it also induced ROP. ROP was first diagnosed in the early 1940s and was named as retrolental fibroplasia previously [5]. Further investigations reported a close association between ROP progression and the high oxygen therapy [6,7,8]. The relationship between supplemental oxygen and ROP was still unclear at that time, but we now understand that the development of ROP is caused by the relative hypoxia after oxygen therapy. Although this supplemental oxygen therapy could significantly reduce the mortality rate of premature infants, ROP emerged as a visual problem globally [9].

ROP is classified as an avoidable and a treatable retinal disease, but the incidence remains high. The latest report about global ROP cases showed that around 190,000 of preterm infants are suffering from any stages of ROP, and 20,000 of them are totally blind or have severe vision impairment [10]. Cryotherapy, laser therapy, and intravitreal anti-vascular endothelial growth factor (VEGF) injection are the current treatments for ROP; however, they may induce the risk of ocular and systemic side effects [11]. Cryotherapy is an effective ablation therapy of abnormal retinal vasculature in ROP, but some unfavorable systemic and functional outcomes may occur [12,13]. On the other hand, laser photocoagulation has better functional outcomes, but it has a higher risk of cataract, myopia, cornea, and lens burns [14,15]. Recently, the intravitreal injection of anti-VEGF agents became a new approach for treating ROP. VEGF is an important growth factor for normal vascular development in infants. However, it also causes abnormal vessel proliferation if its level is extremely high. Indeed, oxidative stress or hypoxia as a result of supplemental oxygen therapy is one of the keys to VEGF upregulation and therefore neovasculature formation during ROP [16]. Since 2011, three different anti-VEGF drugs, bevacizumab, ranibizumab, and aflibercept, have been tested in clinical trials to investigate their efficiency, bioavailability, and functional outcomes for ROP patients [17,18,19,20]. Although the ocular complications such as cataract and retinal scarring were prevented, the systemic VEGF expression is also suppressed after injection, which may then lead to delayed neurodevelopment and impaired neurobehavior in the infants [11,21,22,23]. Taken together, there is a high demand for non-invasive strategies for treating ROP.

Nutraceuticals, or commonly called functional foods, are natural substances that have demonstrated health benefits. The most widespread functional ingredients are polyunsaturated fatty acid (PUFAs), probiotics/prebiotics/symbiotic, and antioxidants [24]. Nutraceuticals such as carotenoids exert anti-oxidative and/or anti-inflammatory responses for preventing diseases such as cardiovascular disease, diabetes, and Alzheimer’s disease [25,26]. Since nutraceuticals are mostly natural plant-based or fruit-based substances, they rarely induce severe adverse effects [27,28] and can be used safely during pregnancy or in children. Breast milk is the most natural nutrient and supplement for the babies. It contains cytokines and growth factors that are beneficial to the infants’ growth [29]. Therefore, maternal intake and thereby supplementing breastmilk or adding supplements to infant formula or food are easy, possible, and non-invasive ways to administer nutraceuticals to the infants.

In vitro and in vivo studies of nutraceutical supplementation to infants have been performed for their protective effects in hypoxia-induced cell injury models or animal ROP models. In this review, we focused on the effect of different types of nutritional oils and herbal nutraceuticals on ROP progression in various models.

## 2. Pathogenesis of ROP

### 2.1. Risk Factors

Understanding the risk factors of ROP is mandatory to determine the directions of disease management and development of predictive models. The major risk factors of ROP include low birth weight, low gestational age, and supplemental oxygen therapy [30]. A previous multicenter study, Cryotherapy for Retinopathy of Prematurity (CYRO-ROP), followed 4099 babies with low birth weight (<1251 g), and it reported that low birth weight and low gestational age are strongly associated with the incidence of ROP [30,31]. Another well-known risk factor of ROP is the use of oxygen therapy. The first concept of supplemental oxygen therapy as a risk factor of ROP was developed in 1956, and it was based on a randomized-controlled trial. It identified that infants exposed to more than 50% oxygen had a higher incidence of ROP [32]. Several recent studies also investigated the association of oxygen concentration and incidence of ROP, including Supplemental Therapeutic Oxygen for Prethreshold Retinopathy of Prematurity (STOP-ROP) and Benefits of Oxygen Saturation Targeting Study II (BOOST-II), but the use of ideal oxygen level remains controversial [9,33,34]. In addition to these three main risk factors, several other risk factors have been discussed recently. They can be divided into maternal, perinatal, and infant factors [30]. Maternal factors mainly stem from the age of the mother, her medication use, and health problems such as hypertension and diabetes. The perinatal factors are commonly associated with the mode of delivery, chorioamnionitis, and premature rupture of membrane. The infant factors consist of gender, multiple birth, lower Apgar score, and hyperglycemia [35,36].

### 2.2. Pathological Vascular Changes in ROP and Its Metabolism

The retina is the most energy-demanding tissue in the human body. A complex and organized retinal vascular network plays the critical role in delivering metabolites, which include oxygen, glucose, and lipid, to the eye [37]. Recently, a specific oxygen-binding protein is identified and named neuroglobin [38,39]. Neuroglobin is highly concentrated in the retina, being 100-fold more when compared with the brain [38]. It is present in all neurons in the retina but not in retinal pigment epithelium (RPE). Neuroglobin acts as an oxygen carrier for transferring oxygen from blood vessels to neurons for satisfying the metabolic needs in mitochondria and thus maintains normal retinal function [40]. In addition to oxygen, glucose and lipids are the other important elements in retinal metabolism. Blood-derived glucose travels across the RPE and the blood–retinal barrier (BRB) and reaches retinal neurons [38]. This process is facilitated by adenosine triphosphate (ATP) and sodium-independent glucose transporters (GLUTs) [41]. Various glucose transporter isoforms are located in the whole retina and play different roles to transfer glucose from choroid capillary to the inner retina. GLUT1 is widely expressed in RPE and photoreceptors for glucose uptake and its metabolism. GLUT2 is present in the Müller cells and is involved in glucose transportation within the retina. GLUT3 is the neuronal GLUT in mammal. It is present in the inner and outer plexiform layers, inner nuclear layer, and ganglion cell layer. Indeed, glycolysis is an important retinal metabolic process, especially in Müller cells. Meanwhile, lactate as a product of glycolysis in Müller cells is released to photoreceptors and other neurons via proton-coupled monocarboxylate transporters (Mct) [38,41]. It can be converted to pyruvate as an energy source for oxidative phosphorylation. Lipid is another mitochondrial fuel for photoreceptors via fatty acid β-oxidation [37]. This process takes place in the mitochondria and peroxisome in mammals. The major fatty acid in retinal energy metabolism is lipids, with more than 16 carbon chains [42,43]. In the mitochondria, fatty acids are broken down, and this generates acetyl-CoA, which can be converted to ATP as energy in the citric acid cycle. In normal retinal metabolism, oxygen, glucose, and lipids delivered from capillaries to the retina provide energy as well as maintain normal retinal functions.

**Figure 1:**
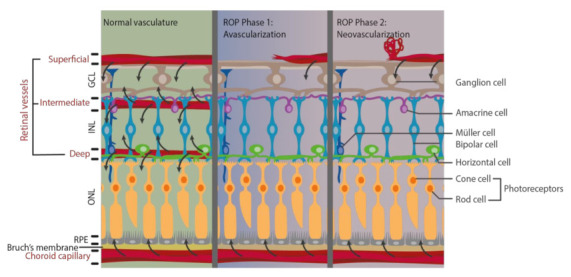
Alteration of metabolic supply after pathological vascular changes during phase 1 and phase 2 of retinopathy of prematurity (ROP). Retinal vasculature is important for delivering oxygen and nutrients to retinal neurons to meet their high metabolic demand (black arrow). However, pathological vascular changes occur in ROP phase 1 and phase 2, leading to inadequate blood flow and thus limit their supply.

Pathological vascular changes in ROP affect nutrient delivery and normal metabolism, which leads to the damage of retinal structure and function (Figure 1). In normal human fetus, retinal vessel development starts at gestational age 16 weeks and expands toward the peripheral retina [11,44]. When the infant is born prematurely, retinal vasculature is still incomplete, and thus, the retina has a peripheral avascular zone. As the cardiopulmonary system is immature, supplemental oxygen therapy is usually provided. However, this hyperoxic environment leads to the first phase of ROP, namely avascularization. Excessive oxygen supply suppresses and interrupts the normal retinal vascular development by inhibiting important angiogenic factors. Hypoxia-inducible factor-1 (HIF-1), VEGF, insulin-like growth factors-1 (IGF-1), nitric oxide (NO), erythropoietin (Epo), and adenosine are downregulated and resulted in interrupted retinal vascular development and retinal avascular zone in phase 1 [16]. The second phase of ROP, neovascularization, begins when the infant returns from supplemental oxygen to room air with a relatively lower oxygen concentration. Oxygen and energy demands cannot be fulfilled when choroid capillary is the only blood supply for metabolism in the avascularized retina [38]. Hypoxia is now generated in phase 2, which stimulates the production of HIF-1, VEGF, IGF-1, NO, Epo, and adenosine. Then, uncontrolled vascular proliferation starts in the avascularized retina by the upregulated angiogenic factors [16,44,45]. Vaso-obliteration in phase 1 of ROP leads to metabolic imbalance under hypoxia in phase 2, which in turns induces pathological neovascularization in ROP.

The regulation of metabolic-related enzymes or hormones may be one way to reduce the severity of ROP. Glycolysis converts glucose to energy under normal glycemic level; however, the polyol pathway reduces glucose to sorbitol and contributes to oxidative stress in hyperglycemic conditions [46]. The deletion of aldose reductase, a first enzyme that is involved in the polyol pathway, showed a beneficial effect on improving retinal function and neuronal responses using a mouse oxygen-induced retinopathy (OIR) model [47,48]. Therefore, an extremely low or high glucose level leads to disease progression by inadequate energy supply or oxidative stress induction [35]. Furthermore, adiponectin is one of the key hormones that modulates glucose and lipid metabolism in insulin-sensitive tissue and plays a role in pathological changes in ROP. The serum adiponectin level in preterm infants is significantly low, especially in the first three weeks of age [49]. Interestingly, recombinant adiponectin treatment improved retinal function in the mouse hyperglycemia-associated retinopathy (HAR) model (see below) [35]. It is suggested that adiponectin modulates glucose metabolism and is able to restore retinal function in the animal ROP model. In addition, interrupting fatty acid β-oxidation by inhibiting the rate-limiting enzyme, carnitine palmitoyltransferase 1, also indicated a vascular protective effect [50]. Future studies are needed to investigate the metabolic modulation in ROP progression.

### 2.3. Animal Models for ROP

Two animal ROP models have been established for studying the two different phases of ROP: HAR for phase 1 and OIR for phase 2.

HAR is a delayed retinal vascular development model by stimulating hyperglycemia in neonates. It was first described in 2018 and is a novel mouse model for demonstrating features observed in ROP phase 1 [35]. Hyperglycemia and low adiponectin level in preterm infants have been shown to be associated with pathological neovascularization in ROP. In HAR, hyperglycemia was induced in mouse neonates. First, 50 mg^−1^ kg^−1^ day^−1^ of streptozotocin is injected into mice from postnatal day (P) 1 to P9 intraperitoneally [35]. Hyperglycemia is stimulated on P8; increased blood glucose and triglyceride levels as well as reduced insulin were observed in P10 mice. Similar to human preterm infants, a low adiponectin level was found in P10 HAR-induced mice. HAR also illustrates the association between glucose metabolism and the adiponectin pathway in ROP. It not only shows the delay of vascular growth in ROP phase 1 but also demonstrates the alteration of retinal metabolism and its related pathways.

OIR is a well-established animal model for proliferative vascular diseases, such as ROP and the late stage of diabetic retinopathy. The principle of this OIR model is the hyperoxic exposure of neonates, leading to vascular obliteration followed by hypoxia when returning to room air, which results in ischemia-induced retinal neovascularization formation. Mouse and rat are common options for animal OIR models due to their postnatal retinal vascular development and the reasonable costs. The murine OIR model was originally established in 1994 [51]. In this model, P7 mouse pups and their mother are placed in an oxygen-controlled chamber (75%) for 5 days and then returned to room air. Central avascularization is formed after hyperoxia, while neovascularization begins on P14, and neovascular formation peaks on P17. As mice can be easily genetically manipulated, investigations on the association of individual genes with pathological neovascularization are feasible using transgenic knockdown or knockout mice in an OIR model. However, OIR in mice cannot fully replicate the vascular changes in human ROP conditions due to the formation of a central avascular area instead of a peripheral one [11,52]. Therefore, the rat OIR model was also generated [53]. A fluctuating oxygen cycle that changes from 50% to 10% oxygen every 24 h was given to P0 to P14 rat neonates. This hyperoxia–hypoxia cycle induces peripheral avascularization and neovascularization in rats, which is similar to human type I severe ROP. Since then, both mouse and rat OIR models have been used for studying the mechanism and drug development in human ROP.

## 3. Nutritional Supplements

**Figure 2:**
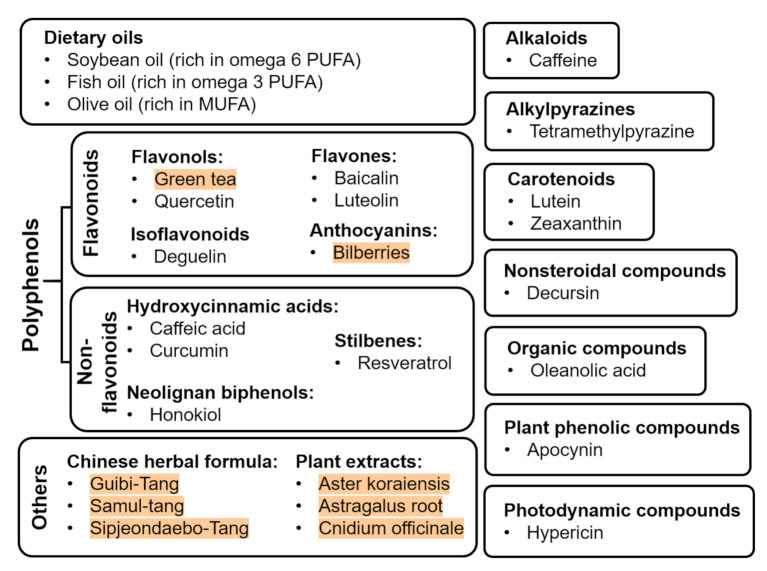
Nutraceuticals with beneficial effects on ROP (plant extracts are highlighted in red).

Abnormal neovascularization in ROP phase 2 is closely associated with metabolic shortage that is caused by the avascularized retina in phase 1. To maintain the metabolic needs in the normal retina, protection of the vasculature is one way to prevent and manage ROP. Providing nutraceuticals is a novel non-invasive strategy to prevent or protect against ROP. In addition to playing a protective role as anti-oxidative, anti-inflammatory, and/or anti-proliferative agents, nutraceuticals can also be provided as a nutritional supplement for postnatal infant growth, which is directly associated with ROP severity [54]. Nutraceuticals can be classified into different classes, such as oils, flavonoids, non-flavonoids, alkaloids, alkylpyrazines, carotenoids, nonsteroidal compounds, organic compounds, photodynamic compounds, plant phenolic compounds, Chinese herbal formula, and other plant extracts (Figure 2). They exert antiangiogenic, antioxidative, and/or anti-inflammatory effects on ROP progression shown in basic translational studies using in vitro and in vivo approaches (Table 1) as well as clinical studies (Table 2).

### 3.1. Oil

**Figure 3:**
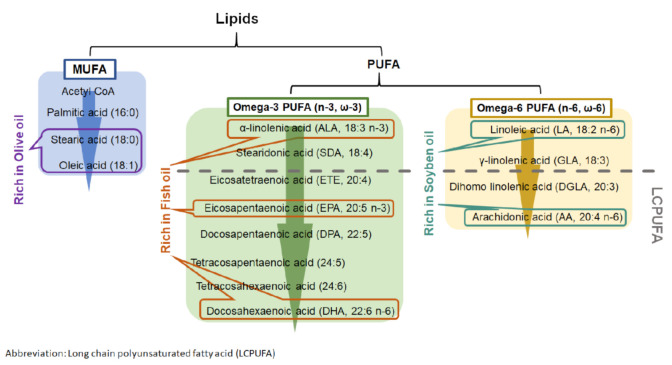
Metabolism of monounsaturated fatty acid (MUFA) and polyunsaturated fatty acid (PUFA).

Lipid emulsions are part of parenteral nutrition for premature infants during their first period of life. They act as the source of energy and fat-soluble vitamins and play a beneficial role in the immune system [85]. Soybean oil (SO), fish oil (FO), and olive oil (OO) are the dietary oils that serve as an oil-based emulsion for immature infants. They contain different combinations of fatty acids that contribute to the formation of phospholipids in cell membranes and the maintenance of its physiological functions and structure as well as photoreceptor differentiation. In addition to their nutritional role, the different nutritional oils can also modulate immune functions. SO-based emulsion is widely used in parenteral nutrition with a high content of omega 6 (n-6 or ω-6) family of PUFA (Figure 3) [85,86]. Although several studies have shown its suppression of immune responses, SO-based emulsion may lead to adverse effects in the unbalanced fatty acid content in cell membrane [87,88,89,90]. In addition to SO, FO also provides protective effects on immunosuppression. FO is the main source of docosahexaenoic acid (DHA), which is critical for cognitive and visual developments in infants [82,86]. Moreover, FO has demonstrated benefits in cardiovascular diseases by suppressing inflammation and oxidation [91]. Recently, several clinical studies showed that the administration of FO–lipid emulsion to very-low-birth-weight (VLBW) infants from the first day of life was beneficial in reducing the risk of developing ROP when compared with those who received SO-based emulsion [82,83]. In addition, FO–lipid supplement in infants increased their serum level of omega 3 (n-3 or ω-3) fatty acids and n-6 fatty acids, such as arachidonic acid (AA), eicosapentaenoic acid (EPA), and DHA [54,92]. OO is another option as a nutritional lipid emulsion for premature infants. OO is rich in monounsaturated fatty acids (MUFAs) and low in PUFAs when compared with FO and SO [86]. Interestingly, OO-based emulsion is well tolerated in preterm infants. It exerts a better anti-oxidative and anti-inflammatory status by lowering lipid peroxidation and suppressing interleukin-6 (IL-6) production when compared with patients who received SO-based emulsion [93,94]. The supplementation of FO, SO, and OO provides different degrees of beneficial effects in anti-oxidation and anti-inflammation.

#### 3.1.1. Polyunsaturated Fatty Acids (PUFAs) and Long-Chain Polyunsaturated Fatty Acid (LCPUFAs)

Dietary PUFAs affect multiple physiological processes in developing infants. Human breast milk contains numerous PUFAs, which include linoleic acid (LA) and α-linoleic acid (ALA). During PUFAs metabolism, LA and ALA can be converted into LCPUFAs with more than 18 carbon atoms (Figure 3). LCPUFAs play a role in visual and behavioral developments in both full term and preterm infants by improving visual acuity and mental development [95,96,97,98,99,100]. As premature infants are at risk in underdevelopment, additional LCPUFAs are required for improving their retinal and cognitive growths.

n-3 and n-6 fatty acids are the essential PUFAs for preterm and VLBW infants and promote normal development and prevent infant morbidity [101]. AA, EPA, and DHA are LCPUFAs that have been investigated as therapeutic agents for ROP. Interestingly, some studies demonstrated that the balance between n-3 and n-6 LCPUFAs can influence immune responses and tissue inflammatory and oxidative responses from hypoxic, oxidative, and inflammatory injuries. The role of n-3 and n-6 LCPUFAs has been demonstrated in clinical trials, in vivo mouse OIR models, and in vitro cell culture studies. n-3 LCPUFA supplementation for preterm infants can promote tissue recovery, prevent damages, and work against ROP [101]. Indeed, n-3 supplementation with 2% DHA and EPA resulted in the suppression of VEGF mediator production, such as NO and IL-1 induced cyclooxygenease-2 (COX-2), inhibition of inflammatory and oxidative responses by downregulating tumor necrosis factor α (TNF-α), intercellular adhesion molecule 1 (ICAM-1) and H_2_O_2_ production, stimulation of adiponectin accumulation, which can inhibit cell proliferation, and result in a reduction of pathological neovascularization [55,61,102,103,104,105,106]. Several studies have shown a lower serum AA level in preterm infants [107,108,109]; in particular, this low AA level in the first month of age in preterm babies has been associated with severe ROP [107], suggesting the importance of AA in vascular integrity and neurovascular connections [54,107]. However, AA supplementation did not significantly halt ROP progression when compared with the use of DHA [61,102,104,105]. Therefore, n-3 LCPUFA supplements such as EPA and DHA appeared to be protective toward ROP in the preterm infants [101]. Yet, due to the suspected complexity in the relationship among various PUFAs, the supplementation of one single type of PUFA may disturb this intricate relationship [54,107]. Indeed, Lofqvist et al. suggested that at present, the supplementation of n-3 PUFAs may be premature [107]. More studies are warranted in the future.

### 3.2. Flavonoids

#### 3.2.1. Green Tea

Green tea promotes anti-oxidative, anti-inflammatory, anti-carcinogenic, and anti-bacterial effects [110,111,112,113]. It is abundant in catechins together with caffeine, vitamins, and minerals. The protective effect of green tea in the eye was first demonstrated in 1999; supplementation of drinking water with green tea suppressed VEGF-mediated corneal neovascularization in mice [114]. Moreover, the impact of green tea was further investigated in a rat OIR model using green tea extract (GTE) and green tea fraction (GTF) [65,66]. GTF is the green tea extract with lower contents of catechins and caffeine with anti-angiogenic effects. OIR-treated rats receiving GTE or GTF treatment responded with inhibition of neovascularization and matrix metalloproteinase-2 (MMP-2) activity. Therefore, a number of animal studies have shown that green tea may promote the suppression of pathological neovascularization and cell proliferation. More information of how green tea affect retinal metabolism via vasculature protection will be needed.

#### 3.2.2. Bilberries

Bilberries (*Vaccinium mytillus*) is a natural source of anthocyanins with high antioxidative properties. Other protective effects, such as anti-inflammatory and anti-apoptotic effects, of bilberries are also reported using murine retinal inflammatory and early age-related macular degeneration models [115]. Moreover, bilberries were shown to inhibit lipid peroxidation and proinflammatory cytokines and to prevent cell apoptosis in a light-induced retinal degeneration model in rabbits [116]. It also prevents retinal leakage and protects against BRB breakdown in an early diabetic retinopathy rat model [117]. Bilberries have anti-oxidative defense properties; therefore, it may play a role in ROP prevention [63]. Bilberry extract was processed, filtered, and concentrated from frozen bilberries. It was further purified by column chromatography and subsequently freeze-dried to powder for storage. One quarter of this purified bilberry extract contains anthocyanins, which include 15 different kinds of anthocyanins. The extract has been intravitreally injected into the mouse eyes on P12 or supplied to human umbilical vein endothelial cells in culture. Both in vivo and in vitro studies showed a reduction of cell proliferation and an inhibition of VEGF-induced phosphorylation of extracellular signal-regulated kinase (ERK) [63]. The suppression of neovascular tufts formation was also observed in the murine OIR model [63]. Therefore, bilberries may prevent ROP progression by protecting retinal vasculature after high oxygen therapy but lack information on neuronal responses and alteration of energy supply after OIR.

#### 3.2.3. Quercetin

Flavonol quercetin is one of the most abundant polyphenols in dietary foods, such as onions, grapes, and berries. It exerts many neuroprotective functions, including anti-oxidation, anti-inflammation, and immunoprotection in in vitro, in vivo as well as clinical studies [118]. It acts as a potent scavenger of reactive oxygen species (ROS) to inhibit cytokine production by astrocytes to reduce inflammatory responses and prevent neurodegenerative diseases such as Alzheimer’s disease and Parkinson’s disease [119,120,121]. Based on these protective roles, quercetin may also reduce the pathological angiogenesis of ROP. A VEGF-induced in vitro cell culture study showed that quercetin can effectively inhibit VEGF and its receptor production, which in turn suppresses endothelial cell proliferation [122,123]. Furthermore, a significant decrease in neovascular area and VEGF expression in the murine OIR model was observed after intraperitoneal injection of 20mg/kg quercetin [68]. However, it may carry toxic effects, such as nephrotoxicity, when administrated for more than 12 weeks or at a high supplemental dose above 1000 mg. Safety studies in animals indicated that quercetin may play a role in estrogen-mediated carcinogenesis and interact with other drugs, which in turn altered their bioavailability [124]. Quercetin can significantly reduce neovascularization in the animal model, but it carries safety issues in high dosage, resulting in long-term adverse effects.

#### 3.2.4. Baicalin

Baicalin is a critical substance that is related to pharmacological actions of a Chinese herbal medicine, *Scutellaria baicalensis*. It has been identified as a compound with anti-inflammatory, anti-oxidative, anti-carcinogenic, and lipid peroxidation preventive effects [125,126,127,128]. It is protective in reducing iron accumulation and ROS production in the rat Parkinson’s disease model and suppressing cytokine production such as IL-6 and TNF-α, thereby inhibiting inflammatory responses in the mouse liver injury model [129,130]. Although it exerts positive effects in oxidative stress and inflammation, its effect on apoptosis was controversial. Baicalin induces apoptosis in different cell lines, including prostate cancer cell line, leukemia-derived T cell line, and human breast cancer cells [131,132,133]. On the other hand, its anti-apoptotic effect was also shown in other models such as the renal ischemia/reperfusion injury model, murine polymicrobial sepsis model, and rat model of permanent focal cerebral ischemia [134,135,136]. When baicalin was injected into mouse pups intraperitoneally in OIR studies, retinal neovascularization was inhibited together with the suppression of VEGF, angiotensin II, and MMP-9 expressions [62], which is suggestive of potent anti-angiogenic effects for ROP prevention.

#### 3.2.5. Luteolin

Luteolin is present in celeries, green peppers, and chamomile tea. Its beneficial neuroprotective effects were investigated, since it was first isolated in 1955 [137]. Numerous studies have shown that luteolin modulates ROS and NO production, inhibits the activities of ERK and TNF-α, as well as blocks cancer cell growth by inhibiting cell proliferation [138,139,140,141,142,143]. Interestingly, it may also have a role in anti-angiogenesis to affect ROP progression [67]. Luteolin injection into mouse neonates on P14 after OIR can suppress neovascularization as well as the migration and tube formation of retinal endothelial cells. It also inhibited ROS production and reduced HIF-1α and VEGF expression [67]. While luteolin plays both anti-oxidative and anti-angiogenic roles in the murine OIR model, more studies on the metabolism-related pathways in the revascularized retina are warranted.

#### 3.2.6. Deguelin

Deguelin is a substance that is derived from *Derris* trifoliata Lour. or *Mundulea* sericea (Leguminosae). It was reported as a natural anti-tumorigenic agent by inducing apoptosis and preventing angiogenesis. Deguelin can effectively decrease the incidence of tumor by suppressing ornithine decarboxylase (ODC), dysregulating the cell-cycle checkpoint protein retinoblastoma to induce apoptosis, inhibiting COX-2 production, and downregulating the Akt pathway in animal models of lung, colon, mammary, and skin cancers [144,145,146,147,148,149,150]. Moreover, its anti-angiogenic effect was well demonstrated by inhibiting HIF-1α and VEGF expressions in various in vitro cancer cell lines, such as human gastric cancer and human breast cancer, and an in vivo mouse tumor model [151,152,153]. This anti-angiogenic characteristic was also described in the mouse laser-induced choroidal neovascularization (CNV) and mouse OIR model [64,154] in which 0.1 µM of deguelin was intravitreal injected 10 days after laser photocoagulation or 2 days after OIR (P14), respectively. These two studies reported the inhibition of tube formation in the CNV model, reduction of neovascularization in the OIR model, and suppression of vascular leakage and HIF-1α-mediated VEGF expression in both models. Although a downregulation of VEGF and HIF-1α by deguelin was described, its impact on the delivery of blood-derived metabolites and neurodevelopment are still unclear.

### 3.3. Non-Flavonoids

#### 3.3.1. Caffeic Acid

Caffeic acid is found in many dietary sources such as coffee, wine, basil, and cabbages. Both in vitro and in vivo studies of caffeic acid have identified its anti-oxidative and anti-inflammatory effects [155,156]. It can significantly reduce ROS, IL-6, and TNF-α production in diabetic mice [157]. Moreover, it contributes to the inhibition of intestinal leakage in rat intestinal ischemia/reperfusion injury and the suppression of lipid peroxidation and DNA damage in in vitro human peripheral blood mononuclear cell culture study [157,158]. In addition to the protective effects in ischemic diseases, it also provides beneficial effects in an OIR model [56]. Caffeic acid applied on human retina microvascular endothelial cells significantly decreased cell proliferation, cell migration, and tube formation in a dose-dependent manner after co-treatment with VEGF. In addition, it also suppressed VEGF expression and ROS production in the endothelial cell culture after H_2_O_2_-induced hypoxia. On the other hand, caffeic acid administrated intravitreally into P14 mouse pups after OIR led to a significant reduction in retinal neovascularization and formation of vascular lumens [56]. Caffeic acid has the potential to treat ROP by preventing neovascular formation with its anti-oxidative characteristics. Meanwhile, more studies on the effects of caffeic acid on retinal lipid metabolism would provide more evidence of its potential benefits.

#### 3.3.2. Curcumin

Curcumin is the major component of turmeric (*Curcumin longa*), which also provides health benefits. Its protective effects were well described in in vitro, in vivo, and clinical studies. Curcumin can modulate inflammation by suppressing nuclear factor-κB (NF-κB) activation, which leads to apoptosis and the suppression of proliferation and downregulates inflammatory cytokine production, including IL-6 and IL-8 [159,160,161,162]. In addition, several animal studies have shown the positive effects of curcumin in inflammatory bowel disease and cancers [163,164,165,166,167,168]. Moreover, numerous clinical trials of curcumin demonstrated its anti-oxidative, anti-inflammatory, and anti-tumorigenic properties in multiple diseases, such as cardiovascular diseases, neurodegenerative diseases, diabetic-related diseases, autoimmune diseases, and ophthalmic and skin disorders [169,170,171,172,173,174,175,176,177,178,179,180,181,182]. In studies related to the eye, curcumin suppressed ROS production and TNF-α release in vitro in the ARPE-19 cell line, in the retinal pigmented epithelial cells, and retinal endothelial cells [183,184]. Although the neuroprotective effects of curcumin were demonstrated in many diseases, it has not shown vascular protective effects in the murine OIR model [69]. Curcumin has been administered by daily intraperitoneal injection after OIR or by a single intravitreal injection on P13. Yet, it could not significantly reduce neovascularization under these two different injection regimens. Curcumin is neuroprotective for many vascular, inflammatory, and neurodegenerative diseases, but it may need another injection protocol to further investigate its protective role in ROP.

#### 3.3.3. Resveratrol

Resveratrol is a natural antioxidant that is abundant in grapes, blueberries, cranberries, peanuts, as well as processed foods such as dark chocolate and red and white wines. Resveratrol was proven to effectively reduce ROS generation and maintain intracellular antioxidant concentration [185,186,187]. Moreover, it provides neuroprotective properties in animal models of stroke, Alzheimer’s disease and Parkinson’s disease by limiting motor impairment and neuronal cell death [185,188,189,190,191,192]. Resveratrol promotes anti-apoptosis, anti-oxidation, and anti-inflammation to protect the retina in the murine light-induced retinal degeneration model and the OIR model [58,70,193]. It can prevent cell death to maintain cell viability and retinal thickness in the hyperoxia-induced retinal primary cell and OIR model, respectively. In addition, it reduces oxidative and inflammatory responses by downregulation of inducible nitric oxide synthase (iNOS), endothelial NOS (eNOS) and neuronal NOS (nNOS) expressions, and inhibition of B-cell lymphoma 2 (Bcl-2) expression. Resveratrol also mediates VEGF expression and suppresses neovascular tuft formation as a result. Indeed, resveratrol can prevent neuronal cell death and may halt ROP by reducing oxidative stress, inflammation, and pathological neovascularization. More investigation on the long-term treatment of resveratrol is needed to study its safety and influences on metabolism, systemic, and visual developments.

#### 3.3.4. Honokiol

Honokiol is a polyphenol isolated from *Magnolia officinalis* and has been shown to have direct anti-cancer and anti-angiogenic roles. It can block the interaction between VEGF and its receptors, inhibit NF-κB that leads to the promotion of apoptosis, and suppress TNF-α and IL-8 expressions, thus downregulating inflammatory responses [194,195,196,197,198]. Honokiol exerts an effective anti-tumor property and demonstrates beneficial effects in numerous preclinical studies utilizing animal models for skin cancer, lung cancer, breast cancer, and prostate cancer [199]. Moreover, the anti-angiogenic role of honokiol was described in both in vitro cell culture study and a murine OIR model [57]. In these studies, honokiol was either administered to cultured human retinal pigment epithelial cells that were placed in a hypoxic chamber or it was intraperitoneally injected into mice after OIR. They showed that honokiol can inhibit the HIF pathway and neovascular tuft formation in both in vitro and in vivo models. Honokiol shows vascular protective properties for OIR, but the effect in neuronal responses and their metabolic pathways are still unclear.

### 3.4. Alkaloids: Caffeine

Caffeine is a common stimulant for the central nervous system and can be obtained from common dietary foods such as coffee, tea, cola, and chocolates. It serves as a blocker of the adenosine A_2A_ receptor. Adenosine is a neuromodulator and inflammatory mediator [200]. The A_2A_ receptor is important for neurodegeneration by the release of NO or other mediators [201]. Caffeine has a beneficial effect on Alzheimer’s disease and Parkinson’s diseases by inactivating the A_2A_ receptor and reducing β-amyloid-induced neurotoxicity [202,203]. Moreover, caffeine has been widely used in neonatology for treating apnea of prematurity since 2006 [204,205]. It can inhibit TNF-α production, reduce the rate of bronchopulmonary dysplasia, and decrease mortality rate without any effects on blood pressure, heart rate, and interruption of brain and renal developments in the preterm infants and extremely preterm infants [206,207,208,209,210,211]. Furthermore, it demonstrated a beneficial effect in a murine OIR. When the water-soluble caffeine was added into drinking water and made available to the mouse pups via maternal breast milk [71], it reduced retinal avascular and neovascular areas, inhibited cell apoptosis, suppressed VEGF production, and downregulated the expression of A_2A_ receptors. Caffeine has been tested in the recent clinical trials of Caffeine for Apnea of Prematurity (CAP) [212,213]. In one of them, Schmidt et al. [212] reported a reduction in ROP with caffeine treatment among other outcomes. However, the authors speculated that this was largely the result of shorter exposure to supplemental oxygen and positive airway pressure. These recent CAP clinical trials also concluded that infants treated with caffeine did not display any delayed neurodevelopment [212,213,214,215,216]. Therefore, caffeine exerts anti-apoptotic, anti-angiogenic, and anti-inflammatory effects in the murine OIR model and did not exert any systemic adverse effects in clinical studies. Nonetheless, more in-depth investigations are essential to determine the influence of metabolic regulation and long-term administration of caffeine as well as health concerns of addiction in preterm infants.

### 3.5. Alkylpyrazines: Tetramethylpyrazine (TMP)

TMP is isolated from the Chinese herbal medicine *Ligusticum wallichii* (*Chuan Xiong*) and serves as a natural plant-derived antioxidant. This herb has been widely used for medicinal purposes in China for over 2000 years [217]. Previously, the actions of TMP were investigated in cardiac and cerebral diseases, but recent studies also indicated its beneficial effects in diabetes, cancers, and liver injury by its anti-oxidative, anti-inflammatory, and anti-apoptotic activities [218,219,220]. It exerts neuroprotective effects by reducing prostaglandin E2 (PGE_2_) production, which is a potent inflammatory mediator, decreasing Bcl-2 expression in cerebral ischemia/reperfusion injury model, suppressing NO and ROS productions in human leukocytes, and protecting against retinal tubular cell death in an ischemia/reperfusion model [221,222,223,224,225]. In addition, the positive effect of TMP has also been demonstrated in the murine OIR model [72]. After daily intraperitoneal injection of 200 mg/kg of TMP, treated mice displayed a reduction in neovascular and avascular formations, downregulation of VEGF and HIF-1α expressions, and a decreased number of apoptotic cells. These studies showed that TMP does not only exert neuroprotective effects in brain injury and ischemic models but also in an animal OIR model. However, no further information about the safety aspects in the use of TMP such as adverse effects or neurodevelopment influences after long-term supplementation was available, which is important when considering its usage in preterm babies.

### 3.6. Carotenoids: Lutein and Zeaxanthin

Lutein and zeaxanthin are xanthophyll carotenoids that are abundant in egg yolk and dark green vegetables. Lutein’s retinal protective role has been examined in in vitro and in vivo models. Lutein maintains cell viability and reduces inflammation after hypoxic damage in in vitro retinal ganglion cells and rat Müller cells [226,227]. Furthermore, the anti-apoptotic and anti-inflammatory properties of lutein were also illustrated by lowering the number of Terminal deoxynucleotidyl transferase-mediated dUTP nick-end labelling (TUNEL)-positive cells and suppressing glial fibrillary acidic protein (GFAP) immunoreactivity in the rat retinal detachment and retinal damage models [228,229]. Lutein can significantly protect from brain damage, leading to better neurological outcomes, and suppress oxidative stress by inhibiting the NF-κB signaling pathway and apoptotic pathway in the mouse ischemia/reperfusion injury model [226,230]. It also prevents tumor formation by the modulation of apoptosis and proliferation, attenuation of oxidative-related damages and induction of cell differentiation [231]. Indeed, lutein has been widely studied in the prevention of stroke, cancers, and eye diseases. Interestingly, lutein and zeaxanthin are unevenly distributed in the human body; they are mainly accumulated in the macula and the lens of the eye. It serves both as an antioxidant and blocker of the high energy blue light by preventing photo-oxidative induced cataract and age-related macular degeneration [11,232]. Although the promising results about the protective effect of lutein were well documented, it remains controversial whether it prevents the incidence of ROP in preterm infants. Lutein supplements have been tested in clinical studies and mouse OIR models. In clinical trials, the addition of around 200 μg lutein or 140 μg lutein and 0.6 μg of zeaxanthin into human milk had shown an ineffective prevention of ROP in the preterm infants [233,234,235]. However, lutein was able to reduce vascular leakage and avascularization and to promote neural protection and revascularization in a mouse OIR model [73]. This discrepancy in lutein effects may be due to lutein’s absorption and bioavailability. As lutein is a lipophilic compound, its absorption and bioavailability are challenging. Recent publications have used oils such as olive oil, soybean oil, corn oil, fish oil, and coconut oil to dissolve lutein for enhancing its bioavailability in mice [236,237]. They found that lutein in olive oil has the highest intestinal absorption and bioavailability in the body. Therefore, the use of lutein and olive oil mixture may be a new direction for investigating the effect of lutein in ROP by increasing in both absorption of lutein and lipid metabolism using olive oil.

### 3.7. Nonsteroidal Compounds: Decursin

Decursin is isolated from the root of *Angelica Gigas*, and it has been shown to mediate cancer formation, angiogenesis, and inflammation [238]. It can effectively suppress tumor growth by inducing apoptosis via G1-phase cell cycle arrest and decreasing proliferation and angiogenesis through VEGF receptor inactivation and protein kinase C activation [239,240,241,242,243,244,245,246]. Meanwhile, decursin has been demonstrated to block COX-2 expression in the inflammatory bowel disease model as well as inhibiting PI3K, ERK, and NF-κB activation in the in vitro cancer cell line studies [247,248,249,250]. Moreover, anti-angiogenic and anti-inflammatory effects were shown in the cell culture study and the murine OIR model [59]. Decursin treatment could inhibit cell proliferation, cell migration, and tube formation in the VEGF-treated human retinal microvascular endothelial cells. A similar result was observed in the in vivo OIR model where decursin reduced neovascularization. In addition, decursin also prevented inflammation by maintaining a homeostatic level of GFAP expression after OIR. Therefore, decursin can promote vascular protection and suppress inflammatory response after OIR, but its vascular-mediated metabolic changes and toxicity are unclear.

### 3.8. Organic Compounds: Oleanolic Acid

Oleanolic acid is a natural medicinal compound commonly found in plants (*Olea europaea*) and the skin or peel of fruits such as lemons, apples, and pears [251,252]. In addition to fruits, it can also be obtained from olives or olive oil. The key pharmacological characteristic of oleanolic acid is its hepatoprotective effects. It is widely used in China as an over-the-counter treatment for liver disorders [252]. Oleanolic acid can significantly accumulate nuclear factor erythroid 2-related factor 2 (Nrf2) inside the nucleus by the activation of PI3K/Akt and ERK signaling pathways and stimulate the downstream Nrf2-related pathway to protect against liver injury [253,254,255,256,257,258]. In addition, it acts as a free radical scavenger to suppress ROS release, upregulates antioxidative enzymes such as thioredoxin peroxidase, and stimulates the production of antioxidant glutathione [259,260,261,262]. Oleanolic acid not only provides hepatoprotective and anti-oxidative properties but also has a positive effect in cancer formation. Oleanolic acid has the ability to induce cell cycle arrest at the G0/G1 phase in in vitro gallbladder cancer cells and prostate cancer cells [263,264]. In an in vivo study, it significantly reduced the initiation of colon cancer in rat [265]. Moreover, oleanolic acid protects the vasculature in the VEGF-mediated proliferative cell culture model and the murine OIR model [60]. It inhibited the VEGF signaling pathway, cell proliferation, and tube formation in the proliferative human umbilical vein endothelial cells. Similarly, neovascularization was suppressed in mice receiving oleanolic acid treatment after OIR. The anti-angiogenic properties of oleanolic acid have been shown; however, studies of inflammatory and oxidative responses after its treatment in both in vivo OIR and in vitro hypoxia models are still lacking.

### 3.9. Photodynamic Compounds: Hypericin

Hypericin is a light-sensitive compound that is isolated from St. John’s wort (*Hypericum perforatum*). It is used as a medicinal herb to treat depression. Other pharmacological activities have also been identified in hypericin, including an anti-cancer effect [266]. Indeed, it is now extensively used in photodynamic therapy for cancer treatments. Hypericin is one of the powerful natural photosensitizers and can accumulate in tumor cells. With the use of a specific wavelength of light, hypericin generates ROS and singlet oxygen, resulting in an induction of oxidative stress and later the necrosis and apoptosis of tumor cells [266,267,268,269,270,271,272]. The function of hypericin can be altered by different light doses. Low light doses induce angiogenesis and cell survival by activating the c-Jun N-terminal protein kinase 1 (JNK1) and p38α pathways. The use of a medium light dose leads to apoptosis by releasing cytochrome C while the high light dose stimulates necrosis [266]. The antiviral function of hypericin was also demonstrated, but its clinical use is still controversial. Several studies have demonstrated the inhibition of reproduction and reduction of adsorptive ability in Hand-foot-and-mouth disease and respiratory syndrome; however, no antiviral effect was observed in HIV-1 and hepatitis C virus patients after hypericin administration [273,274,275]. The anti-angiogenic effect of hypericin was also observed in the mouse OIR model [75]. When hypericin and St. John wort were administered to mouse pups, both effectively suppressed neovascular formation and inhibited the VEGF-mediated pathway by downregulating phosphorylated ERK expression. Hypericin has the ability to significantly reduce pathological neovascularization after OIR; however, there is still inadequate information on its anti-inflammatory and anti-oxidative roles, metabolic changes, and long-term safety problems in animal OIR or hypoxia-related cell culture studies.

### 3.10. Plant Phenolic Compounds: Apocynin

Apocynin is a plant-derived medical compound that can be isolated from a variety of plants such as roots of *Picrorhiza kurroa*. Nicotinamide adenine dinucleotide phosphate (NADPH) oxidase inhibition is a well-known property of apocynin. NADPH oxidase is activated by binding its subunits including membrane-bound subunit p22^phox^ and cytoplasmic subunit p47^phox^, leading to ROS and high active hydroxyl radical generation. Apocynin is a powerful inhibitor by interrupting the interaction between p22^phox^ and p47^phox^ [276]. Moreover, apocynin acts as a peroxisome proliferator-activated receptor gamma (PPARγ) agonist that can suppress the expression of NADPH oxidase in turn to reduce oxidative stress [277,278]. In addition to its role as an antioxidant, apocynin can also be used to treat autoimmune disease and neurodegenerative diseases. One example is the autoimmune inflammatory disease such as rheumatoid arthritis when the oral administration of apocynin restored cartilage proteoglycan production in the arthritic joint [279]. In addition, apocynin mediates microglial-mediated neurotoxicity in the brain and might be used in patients with Alzheimer’s disease and Parkinson’s disease. Diseases-associated microglia regulates the activation of NADPH oxidase and produces ROS. Recent clinical studies have shown that Alzheimer’s disease patients frequently have high levels of neurotoxic agents such as ROS, NO, and TNF-α, which are secreted from disease-associated microglia and NADPH oxidase in their brains [280,281,282]. Apocynin can suppress NADPH oxidase by re-balancing the level between proinflammatory microglia and homeostatic microglia by downregulating the production of IL-1β, TNF-α, and NO [283,284]. In addition, the potential protective role of apocynin in Alzheimer’s disease was further studied in a mouse model. After apocynin treatment, the size of the plaques within the cortex and hippocampus as well as the microglia number in the cortex were significantly reduced, but it is ineffective to improve the neurological outcomes [285]. Although the use of apocynin is still unclear in Alzheimer’s disease, it is shown to promote anti-oxidative and anti-angiogenic effects in the OIR model [74]. Apocynin not only prevented neovascularization and reduced avascular zone and VEGF level but also inhibited oxidative stress and suppressed activated p47^phox^ accumulation. These data support the idea that apocynin can indirectly suppress ROP progression by inhibiting NADPH oxidase and activating PPARγ. There is still a need to investigate its safety profile, since it can reduce VEGF levels and may therefore also induce other systemic or neurodevelopmental problems in infants.

### 3.11. Chinese Herbal Formulas and Other Plant Extracts

Herbal formulas and plant extracts may also exhibit anti-angiogenic effects in the OIR models, although the mechanism is not fully understood. The use of Chinese medicine or Chinese herbal formula can be traced back to around 2200 years ago. Chinese herbal formula is a mixture of different medicinal herbs, animals, or minerals that are made into a powder, pill, or liquid form after boiling. Several of them have been standardized, and guidelines are provided for the usage of the formula. Guibi-Tang, Samul Tang, and Sipjeondaebo-Tang are the standardized Chinese formula that have a vascular protective role in an OIR model [79,80,81]. They act as anti-angiogenic agents to directly inhibit VEGF production and reduce avascular and neovascular areas after treatments. Other plant extracts including *Aster koraiensis* extract, *Astragalus* root extract, and *Cnidium officinale* extract also exert beneficial effects after OIR, resulting in vascular protection and the inhibition of angiogenic agents release [76,77,78]. It is important to remember that these are plant extracts but not a pure compound and thus contain other compounds that are present inside the plant. Although they display a significant decrease of pathological neovascular formation after treatment, the principle behind is unclear, and there are also safety concerns for usage in infants.

## 4. Conclusions and Future Perspectives

In the past 20 years, the administration of nutraceuticals has been proposed for the prevention and management of ROP. ROP is an eye disease commonly present in preterm infants who suffer from immature neuronal, vascular, organ, and immune developments. Pathological angiogenesis in ROP affects blood supply to the retina as well as induces energy and oxygen shortage and disrupts retinal function. The suppression of abnormal neovascular formation will modulate and restore retinal metabolism and hence prevent ROP. However, current treatments for ROP patients are invasive and may induce unfavorable outcomes such as delayed neurodevelopment. Nutraceuticals are natural substances; they can be administered to infants in non-invasive routes such as breast milk or infant formulas. Another route worthy of consideration is the use of nutraceuticals in pregnant mothers, which is potentially better and safer, but this needs further investigation. Although numerous functional dietary oils and plant-derived compounds display neuroprotective and vascular protective roles in different oxidative, inflammatory, and/or neurodegenerative diseases, not all of them are protective against ROP.

Among the many studies that demonstrate the benefits of nutraceuticals in preventing pathological neovascularization in different in vivo animals and in vitro proliferative- and hypoxia-induced models, only a limited number investigated the underlying mechanisms of action. Most of them indicated a suppression of VEGF or angiogenesis-related factors by nutraceuticals, which include bilberry extract, quercetin, baicalin, luteolin, deguelin, caffeic acid, resveratrol, honokiol, caffeine, TMP, decursin, oleanolic acid, hypericin, and apocynin. In addition, some of the nutraceuticals, such as DHA, resveratrol, TMP, lutein, decursin, and apocynin, also exert anti-oxidative, anti-inflammatory, or anti-apoptotic effects in these studies. However, the mechanisms of action of some nutraceuticals that are potentially beneficial in ROP-related studies are not discussed, despite their roles in anti-angiogenesis, anti-oxidation, and anti-inflammation in other disease models. Among the various proposed mechanisms of action, we hypothesized that anti-angiogenesis appeared to be the most common one for nutraceuticals so far. Nonetheless, more studies to confirm their mechanisms of action are warranted.

Moreover, the safety issues for the long-term administration of nutraceuticals have not been explored. From a clinical perspective, one anti-VEGF injection for ROP treatment is usually insufficient; the addition of nutraceuticals could be effective in extending the effect. Then, additional injections could be omitted, and the anti-VEGF systemic load could be reduced. Additional laser treatment might also become unnecessary. Yet, before nutraceuticals can be considered as therapeutic agents or adjuncts for ROP infants in a natural and non-invasive approach, studies should be conducted to investigate their functional, neuronal, and behavioral outcomes, bioavailability, means of delivery such as nanoformulation, and safety aspects associated with long-term consumption, as well as the underlying mechanism in treating ROP.

## Figures and Tables

**Table 1:** Effects of nutraceutical supplement in the basic studies of ROP-related models.

	Family of Compound	Dietary Source/Compound	Dosage	Supplementation Period	Cell Culture/Animal Model	Effects	Reference(s)
**In vitro**	Dietary oil	Docosahexaenoic acid (DHA)	0.5 to 10 μM	/	Retinal ganglion cell (RGC-5) (H_2_O_2_ induced hypoxia)	Anti-apoptosis Increased cell viability after treatment	[55]
	Non- flavonoids	Caffeic acid	10 to 200 μM	/	Human retina microvascular endothelial cells (VEGF-induced proliferation)	Anti-angiogenesis Inhibition of cell proliferation Inhibition of cell migration and tube formation (100 μM)	[56]
			100 μM	/	Human retina microvascular endothelial cells (H_2_O_2_-induced hypoxia)	Anti-angiogenesis Suppression of VEGF expression Anti-oxidation Reduction of ROS production	
		Honokiol	20 μM	/	Human retinal pigment epithelial cell lines (Hypoxic chamber)	Anti-angiogenesis Suppression of HIF expression	[57]
		Resveratrol	5 to 100 μg/ml	/	Primary culture of dissociated-dispersed retinal cells (Hyperoxia induction by 100% O2 incubation for 6 h)	Anti-inflammation Suppression of iNOS, eNOS and nNOS expression Anti-apoptosis Maintain cell viability comparing with control group	[58]
**In vitro**	Nonsteroidal compounds	Decursin	1 to 50 μM	/	Human retinal pigment epithelial cell lines (VEGF-induced proliferation)	Anti-angiogenesis Suppression of VEGF receptors’ expression Reduction of tube formation, cell proliferation and cell migration Maintained the cell viability	[59]
	Organic compounds	Oleanolic acid	0.1 to 50 μM	/	Human umbilical vein endothelial cells (VEGF induced proliferation)	Anti-angiogenesis Inhibition of VEGF signaling pathway Suppression of cell proliferation and tube formation (10 μM)	[60]
**In vivo**	Dietary oils	2% ω-3 LCPUFAs (1% DHA and 1% EPA) or 2% ω-6 LCPUFAs (AA)	10% (w/w) safflower oil containing either 2% ω-3 LCPUFAs (1% DHA and 1% EPA) or 2% ω-6 LCPUFAs (AA) (Oral)	P1 to P17	Mouse OIR model (75% O_2_, P7 to P12)	Anti-angiogenesis Reduction of AV and NV areas	[61]
	Flavonoids	Baicalin	1 or 10 mg/kg/day (Intraperitoneal)	P12 to P17	Mouse OIR model (75% O_2_, P7 to P12)	Anti-angiogenesis Suppression of VEGF, angiotensin II, and MMP-9 expression Reduction of NV and AV areas	[62]
		Bilberry extract	300 ng per eye (Intravitreal)	P12	Mouse OIR model (75% O_2_, P7 to P12)	Anti-angiogenesis Inhibition of cell proliferation Inhibition of VEGF induced phosphorylation of ERK 1/2 and Akt Reduction of NV and AV areas	[63]
**In vivo**	Flavonoids	Deguelin	0.1 μM (Intravitreal)	P14	Mouse OIR model (75% O_2_, P7 to P12)	Anti-angiogenesis Suppression of VEGF and HIF-1α expression Inhibition of vascular leakage Reduction of NV area	[64]
		Green tea extract	12.5% or 25% GTE (Oral)	P6 to P17	Rat OIR model (P0 to P12)	Anti-angiogenesis Reduction of NV and AV areas	[65]
		Green tea fraction (With less content of catechins and caffeine)	0.01 or 0.05 g/mL/day GTF (Oral)	P6 to P17	Rat OIR model (P0 to P12)	Anti-angiogenesis Reduction of NV and AV areas	[66]
		Luteolin	0.1 or 10 μM (Intravitreal)	P14 to P17	Mouse OIR model (75% O_2_, P7 to P12)	Anti-angiogenesis Suppression of VEGF and HIF-1α expression Reduction of NV area Anti-oxidation Reduction of ROS production	[67]
		Quercetin	20 mg/kg (Intraperitoneal)	P12 to P17	Mouse OIR model (75% O_2_, P7 to P12)	Anti-angiogenesis Suppression of VEGF expression Reduction of NV area	[68]
	Non-flavonoids	Caffeic acid	100 μM (Intravitreal)	P14	Mouse OIR model (75% O_2_, P7 to P12)	Anti-angiogenesis Reduction of NV area and vascular lumen formation	[56]
**In vivo**	Non- flavonoids	Curcumin	50 or 100 mg/kg/day (Intraperitoneal)	P12 to P17	Mouse OIR model (75% O_2_, P7 to P12)	No significant difference in the number of vascular lumens between treated group and control group	[69]
			0.1 or 1 μg (Intravitreal)	P13			
		Honokiol	10–20 mg/kg/day (Intraperitoneal)	P12 to P17	Mouse OIR model (75% O_2_, P7 to P12)	Anti-angiogenesis Reduction of NV area	[57]
		Resveratrol	30 mg/kg/day (Intravitreal)	P14 to P21	Rat OIR model (P1 to P14)	Anti-inflammation Suppression of iNOS, eNOS, and nNOS expression Anti-apoptosis Maintain retinal thickness comparing with control group	[58]
			10, 30, or 60 mg/kg/day (intragastrical)	P12 to P17	Rat OIR model (P7 to P12)	Anti-angiogenesis Suppression of VEGF expression Anti-inflammation and oxidation Suppression of Bcl-2 expression	[70]
	Alkaloids	Caffeine	0.1, 0.3, and 1 g/L in drinking water for lactating mothers (uptake via maternal breast milk)	P0 to P17,P7 to P12, orP12 to P17	Mouse OIR model (75% O_2_, P7 to P12)	Anti-angiogenesis Reduction of NV and AV areas Suppression of VEGF expression Anti-apoptosis Reduced TUNEL-positive cells Anti-inflammation Inhibition of A_2A_ receptor expression	[71]
**In vivo**	Alkylpyrazines	Tetramethylpyrazine	200 mg/kg/day	P12 to P17	Mouse OIR model (75% O_2_, P7 to P12)	Anti-angiogenesis Reduction of NV and AV areas Suppression of VEGF and HIF-1α expressions Anti-apoptosis Reduced TUNEL positive cells Prevented the loss of neurons Maintained normal morphology of retina Anti-inflammation Preserved astrocyte template Suppression of GFAP expression	[72]
	Carotenoids	Lutein and Zeaxanthin	0.2 mg/kg/day (Intraperitoneal)	P12 to P17	Mouse OIR model (75% O_2_, P7 to P12)	Anti-angiogenesis Reduction of AV area Prevention of retinal blood vascular leakage Facilitated endothelial cell tip cell formation Anti-inflammation Preserved astrocytic template Maintained glial and microglial cell responses (comparing with control group)	[73]
	Nonsteroidal compounds	Decursin	5 µM (Intravitreal)	P14	Mouse OIR model (75% O_2_, P7 to P12)	Anti-angiogenesis Reduction of NV area Anti-inflammation Suppression of GFAP expression	[59]
**In vivo**	Organic compounds	Oleanolic acid	62.5 or 125 mg/kg (Intraperitoneal)	P11 and P15	Mouse OIR model (85% O_2,_ P8 to P11)	Anti-angiogenesis Reduction of NV area	[60]
	Plant phenolic compounds	Apocynin	10 mg/kg/day (Intraperitoneal)	P12 to P17	Rat OIR model (P0 to P14)	Anti-angiogenesis Reduction of NV and AV areas Suppression of VEGF expression Anti-oxidation Suppressed oxidative stress Reduced phosphorylation of the subunit of NADPH oxidase	[74]
	Photodynamic compounds	Hypericin	15 mg/kg/day of St. John Wort (Oral)	P12 to P17	Mouse OIR model (75% O_2_, P7 to P12)	Anti-angiogenesis Reduction of NV and AV areas Suppression of VEGF-mediated pathway	[75]
			15, 45, and 135 µg/kg/day of hypericin (Oral)				
	Plant extracts	*Aster koraiensis* Extract	25 or 50 mg/kg/day (Intraperitoneal)	P12 to P16	Mouse OIR model (75% O_2_, P7 to P12)	Anti-angiogenesis Reduction of NV area Suppression of VEGF expression	[76]
		*Astragalus* root extract	10, 20 or 40 mg/kg/day (Intragastrical)	4 weeks	Mouse OIR model (75%O_2_)	Anti-angiogenesis Reduction of vascular leakage Suppression of VEGF and HIF-1α expression Anti-apoptosis Reduced TUNEL-positive cells Anti-oxidation Inhibition of ROS production	[77]
**In vivo**	Plant extracts	*Cnidium officinale* extract	100 mg/kg/day (Intraperitoneal)	P12 to P16	Mouse OIR model (75% O_2_, P7 to P12)	Anti-angiogenesis Reduction of NV and AV areas Suppressed expression of VEGF and angiogenic-related factors	[78]
	Chinese herbal formula	Guibi-tang	50 or 100 mg/kg/day (Intraperitoneal)	P12 to P16	Mouse OIR model (75% O_2_, P7 to P12)	Anti-angiogenesis Reduction of NV and AV areas Suppressed expression of VEGF and angiogenic-related factors	[79]
		Samul-tang	10 or 50 mg/kg/day (Intraperitoneal)	P12 to P16	Mouse OIR model (75% O_2_, P7 to P12)	Anti-angiogenesis Reduction of NV and AV areas Suppressed expression of VEGF and angiogenic-related factors	[80]
		Sipjeondaebo-tang	50 or 100 mg/kg/day (Intraperitoneal)	P12 to P16	Mouse OIR model (75% O_2_, P7 to P12)	Anti-angiogenesis Reduction of NV area Suppression of VEGF expression	[81]

**Table 2:** Effects of nutraceutical supplement in the clinical studies of ROP.

Family of compound	Dietary Source/Compound	Dosage	Supplementation Period	Candidates for Clinical Study	Effects	Reference(s)
**Dietary oils**	20% Clinoleic: 50% of soybean and olive oil emulsion 10% Omegaven: 50% of fish-oil emulsion	(Intravenous, Daily) Birth weight of <1000 g: 0.15 g of Omegaven and 0.35 g of Clinoleic Birth weight of >1000 g: 0.35 g of Omegaven and 0.65 g of Clinoleic (Maximum: 1.0–1.2 g of Omegaven and 2.0–2.3 g of Clinoleic)	From the first day of life	Clinical, preterm infants <32 weeks’ gestational age, weighed <1250 g	Reduction of ROP incidence Reduced risk of laser therapy for infants	[82]
	20% Intralipid: soybean oil 200 g/dL20% SMOFlipid: soybean oil 60 g/dL, MCT 60 g/dL, olive oil 50 g/dL, fish oil 30 g/dL	(Intravenous, Daily) Initial dose: 0.5 g of lipids per kg of body weight (birth weight of <1000 g) or 1.0 g of lipids per kg of body weight (birth weight of >1000 g) (Maximum of 3.0 g of lipids per kg of body weight/day)	From the first day of life	Clinical, preterm infants <32 weeks’ gestational age, weighed <1250 g	Reduction of ROP incidence (in 20% SMOF lipid treated group)	[83]
	20% Lipovenoes medium-chain triglycerides: 50% soybean oil SMOFlipid: 30% soybean oil, 25% olive oil, and 15% fish oil	3 g/kg body weight/day (Intravenous)	Within 24 h of birth for a duration of at least 7 consecutive days	Clinical, preterm infants weighed <1500 g	Reduction of ROP incidence (in SMOFlipid treated group) Reduced the need of anti-VEGF treatment	[84]

## Data Availability

Exclude.

## Abbreviations

AA	Arachidonic acid
ALA	α-linoleic acid
ATP	Adenosine triphosphate
AV	Avascular
Bcl-2	B-cell lymphoma 2
BRB	Blood–retinal barrier
CNV	Choroidal neovascularization
COX-2	Cyclooxygenease-2
DHA	Docosahexaenoic acid
ERK	Extracellular signal-regulated kinase
eNOS	Endothelial nitric oxide synthase
EPA	Eicosapentaenoic acid
Epo	Erythropoietin
FO	Fish oil
GFAP	Glial fibrillary acidic protein
GLUTs	Sodium-independent glucose transporters
HAR	Hyperglycemia-associated retinopathy
HIF-1	Hypoxia-inducible factor-1
ICAM-1	Intercellular adhesion molecule 1
IGF-1	Insulin-like growth factors-1
IL-6	Interleukin-6
iNOS	Inducible nitric oxide synthase
JNK1	c-Jun N-terminal protein kinase 1
LA	Linoleic acid
MMP-2	Matrix metalloproteinase-2
MUFAs	Monounsaturated fatty acids
n-3	Omega 3
n-6	Omega 6
NADPH	Nicotinamide adenine dinucleotide phosphate
NF-κB	Nuclear factor-κB
nNOS	Neuronal nitric oxide synthase
NO	Nitric oxide
Nrf2	Nuclear factor erythroid 2-related factor 2
NV	Neovascular
ODC	Ornithine decarboxylase
OIR	Oxygen-induced retinopathy
OO	Olive oil
PGE2	Prostaglandin E2
PPARγ	Peroxisome proliferator-activated receptor gamma
PUFAs	Polyunsaturated fatty acids
ROP	Retinopathy of prematurity
ROS	Reactive oxygen species
RPE	Retinal pigment epithelium
SO	Soybean oil
TNF-α	Tumor necrosis factor α
TUNEL	Terminal deoxynucleotidyl transferase-mediated dUTP nick-end labeling
VEGF	Vascular endothelial growth factor
